# Prediction Aided Tapering In rheumatoid arthritis patients treated with biOlogicals (PATIO): protocol for a randomized controlled trial

**DOI:** 10.1186/s13063-022-06471-x

**Published:** 2022-06-16

**Authors:** Marianne A. Messelink, Matthijs S. van der Leeuw, Alfons A. den Broeder, Janneke Tekstra, Marlies C. van der Goes, Marloes W. Heijstek, Floris Lafeber, Paco M. J. Welsing

**Affiliations:** 1 Department of Rheumatology & Clinical Immunology, University Medical Center Utrecht, Utrecht University, Heidelberglaan 100, 3508 GA Utrecht, The Netherlands; 2 Department of Rheumatology, Sint Maartenskliniek, Hengstdal 3, 6574 NA Ubbergen, The Netherlands; 3 Department of Rheumatology, Meander Medical Center, Maatweg 3, 3813 TZ Amersfoort, The Netherlands

**Keywords:** Rheumatoid arthritis, Dose reduction, Tapering, bDMARD, Biological, Clinical decision aid, Prediction model, Randomized controlled trial, Study protocol, Predictive algorithm

## Abstract

**Background:**

Biological disease-modifying anti-rheumatic drugs (bDMARDs) are effective in the treatment of rheumatoid arthritis (RA) but are expensive and increase the risk of infection. Therefore, in patients with a stable low level of disease activity or remission, tapering bDMARDs should be considered. Although tapering does not seem to affect long-term disease control, (short-lived) flares are frequent during the tapering process. We have previously developed and externally validated a dynamic flare prediction model for use as a decision aid during stepwise tapering of bDMARDs to reduce the risk of a flare during this process.

**Methods:**

In this investigator-initiated, multicenter, open-label, randomized (1:1) controlled trial, we will assess the effect of incorporating flare risk predictions into a bDMARD tapering strategy. One hundred sixty RA patients treated with a bDMARD with stable low disease activity will be recruited. In the control group, the bDMARD will be tapered according to “disease activity guided dose optimization” (DGDO). In the intervention group, the bDMARD will be tapered according to a strategy that combines DGDO with the dynamic flare prediction model, where the next bDMARD tapering step is not taken in case of a high risk of flare. Patients will be randomized 1:1 to the control or intervention group. The primary outcome is the number of flares per patient (DAS28-CRP increase > 1.2, or DAS28-CRP increase > 0.6 with a current DAS28-CRP ≥ 2.9) during the 18-month follow-up period. Secondary outcomes include the number of patients with a major flare (flare duration ≥ 12 weeks), bDMARD dose reduction, adverse events, disease activity (DAS28-CRP) and patient-reported outcomes such as quality of life and functional disability. Health Care Utilization and Work Productivity will also be assessed.

**Discussion:**

This will be the first clinical trial to evaluate the benefit of applying a dynamic flare prediction model as a decision aid during bDMARD tapering. Reducing the risk of flaring during tapering may enhance the safety and (cost)effectiveness of bDMARD treatment. Furthermore, this study pioneers the field of implementing predictive algorithms in clinical practice.

**Trial registration:**

Dutch Trial Register number NL9798, registered 18 October 2021, https://www.trialregister.nl/trial/9798. The study has received ethical review board approval (number NL74537.041.20).

**Supplementary Information:**

The online version contains supplementary material available at 10.1186/s13063-022-06471-x.

## Administrative information

Note: the numbers in curly brackets in this protocol refer to SPIRIT checklist item numbers. The order of the items has been modified to group similar items (see http://www.equator-network.org/reporting-guidelines/spirit-2013-statement-defining-standard-protocol-items-for-clinical-trials/).

**Table:** 

Title {1}	Prediction Aided Tapering in rheumatoid arthritis patients treated with bIOlogicals (PATIO): Protocol for a randomized controlled trial
Trial registration {2a and 2b}.	Dutch trial registry number NL9798
Protocol version {3}	Protocol version 1.0 (September 2021)
Funding {4}	This study is funded by a research grant from The Netherlands Organisation for Health Research and Development (ZonMW). Grant number: 848018009.
Author details {5a}	*Department of Rheumatology & Clinical Immunology, University Medical Center Utrecht (UMCU)* drs. M. A. Messelink, drs. M. van der Leeuw, dr. J. Tekstra, prof. F. Lafeber, dr. P.M.J. Welsing, dr. M.W. Heijstek *Department of Rheumatology, Meander Medical Center, Amersfoort, The Netherlands* dr. M.C. van der Goes *Department of Rheumatology, Sint Maartenskliniek, Ubbergen, The Netherlands* dr. A.A. den Broeder
Name and contact information for the trial sponsor {5b}	Sponsor: University Medical Center Utrecht (UMCU), Heidelberglaan 100, 3584 CX, Utrecht, The Netherlands. Postal address: G02.228, P.O. Box 85,500, 3508GA, Utrecht, The Netherlands.
Role of sponsor {5c}	This is an investigator-initiated trial by the UMCU. The UMCU is responsible for the study design; collection, management, analysis and interpretation of data; writing of the report; and the decision where to submit the report for publication. The UMCU will have ultimate authority over these activities.

## Introduction

### Background and rationale {6a}

Biological disease-modifying anti-rheumatic drugs (bDMARDs) are effective in the treatment of rheumatoid arthritis (RA), improving clinical, functional, and radiographic outcomes [1]. Examples of bDMARDs include infliximab, adalimumab, etanercept, abatacept, and tocilizumab. Many RA patients who are treated with bDMARDs achieve long periods of stable low disease activity or remission [2]. However, bDMARDs may also lead to adverse events, call for self-injections, or hospital visits and are expensive [3–5]. Thus, tapering bDMARDs to the lowest effective dose is of great clinical interest and may support the sustainability of the healthcare system as a whole.

The most successful and cost-effective strategy for tapering appears to be “disease activity-guided dose optimization” (DGDO) [6–8]. This means the dose is gradually tapered, until either disease activity flares or the bDMARD is discontinued. Two randomized trials have demonstrated that, using this strategy, 63–80% of patients can taper or even stop their bDMARD [6, 7]. No important differences were observed in the proportion of patients with low disease activity or remission after 18 months between DGDO and usual care [7].

However, since DGDO is a ‘trial and error’ approach, flares occur frequently during the tapering process, for which the previously effective dose needs to be reinstated or additional therapy is necessary. Although these short-lived flares do not seem to relevantly affect radiographic progression or long-term disease activity, there is conflicting evidence regarding functional outcome and impact on quality of life [7, 9]. Therefore, it would be beneficial to predict whether, and to which extent, a bDMARD can be tapered in a particular patient without a flare occurring.

For this purpose, we recently developed and externally validated a dynamic flare prediction model [10, 11]. The model combines both fixed and longitudinal patient and disease characteristics to predict the risk of a flare at every visit to the outpatient clinic. In case of a high predicted risk, tapering can be halted in time to prevent a flare. We simulated the incorporation of the prediction model in a DGDO tapering strategy in data of the DRESS trial, which significantly reduced flares while maintaining bDMARD dose reduction [7, 11]. In the presented randomized controlled trial (RCT), we will evaluate if these promising results can be confirmed in clinical practice. In this paper, we will discuss the design of this RCT and its rationale.

### Objectives {7}

Our aim is to assess whether incorporating dynamic flare risk predictions into a bDMARD tapering strategy can reduce the number of flares while maintaining bDMARD dose reduction in RA patients with a stable low level of disease activity.

### Trial design {8}

Pragmatic, open, randomized, superiority, multi-center strategy trial with 18 months follow-up. Patients will be randomized in a 1:1 ratio.

## Methods: participants, interventions, and outcomes

### Study setting {9}

Patients will be recruited through outpatient rheumatology clinics of participating centers in the Netherlands. Currently, we aim at participation of seven centers in the Netherlands in this trial.

### Eligibility criteria {10}

#### Inclusion criteria

- A clinical diagnosis of rheumatoid arthritis as assessed by the treating rheumatologist
- Compliance with (subcomponents of) the ACR 1987 or EULAR/ACR 2010 criteria will be reported
- Treatment of their RA with one of the following bDMARDs in ≥ 66% (i.e., maximally one dose reduction step previously taken) of the defined daily dose based on the Dutch pharmacological guidelines [12]: adalimumab, certolizumab, golimumab, infliximab, etanercept, sarilumab, tocilizumab or abatacept, either as monotherapy or in combination with a conventional synthetic (cs)DMARD
- Patient is eligible to taper bDMARD according to the treating physician (e.g., no other indication for bDMARD such as psoriasis, no recent relevant radiographic progression)
- Stable low disease activity (LDA) on current bDMARD for ≥ 6 months according to treating physician
- Current stable low disease activity according to treating physician *and* a maximum DAS28-CRP of 3.5 (i.e., the cut-off point for low disease activity of 2.9 + measurement error in DAS28 (~ 0.6) [13–15])
- Patient is willing to taper (and if possible, stop) his/her bDMARD as well as to continue his/her current bDMARD dose
- At least 18 years of age and consenting

#### Exclusion criteria

- Recent earlier (< 6 months) tapering attempt(s) with the same bDMARD that failed according to treating physician
- Inability to comply with protocol, e.g., no possibility to measure outcome over 18 months, insufficient knowledge of the Dutch language

The inclusion and exclusion criteria aim to resemble the conditions in which bDMARD tapering would be considered in clinical practice. When both the patient and physician are satisfied with the level of disease activity and do not wish to intensify, maintaining this level of disease activity with less medication is of added clinical value. Therefore, if a patient has a DAS28-CRP slightly above the DAS28-CRP low disease activity (LDA) threshold of 2.9, but the treating physician and the patient judge that there is a stable low level of disease activity, the patient is still eligible for inclusion. This is also in line with the DRESS study [7], the fact that the DAS28-score has a measurement error of around 0.6 [13], and the possibility of overestimation of the DAS28-score in patients with comorbidities such as fibromyalgia [16].

If patients do not meet the ACR or EULAR/ACR diagnostic criteria, the individual components of these criteria will be registered to enable exploring this subgroup in more detail in later analysis. Furthermore, we have chosen not to include patients treated with rituximab, due to the long (often ≥ 6 months) dosing intervals of this bDMARD.

### Who will take informed consent? {26a}

Patients will be approached for participation by their treating physician at the rheumatology department. In case a patient is interested to participate, a research physician or nurse will inform the patient and if appropriate obtain informed consent.

### Additional consent provisions for collection and use of participant data and biological specimens {26b}

We will also ask patients for permission to approach them for future related research.

### Interventions

#### Explanation for the choice of comparators {6b}

The aim of the current RCT is to assess whether the incorporation of flare risk predictions in a bDMARD tapering strategy can reduce the number of flares during tapering, while preserving the tapering potential as optimal as possible. For this purpose, it is necessary to compare the current optimal tapering strategy with and without the incorporation of the results of a flare prediction model. The most successful and cost-effective strategy for tapering appears to be DGDO [6–8]. This. is a treat-to-target strategy, in which the bDMARD dose is gradually tapered, until either disease activity flares or the bDMARD is discontinued. During this process, disease activity is monitored closely to enable a swift increase in bDMARD dose when a flare occurs, in order to quickly resolve the flare. In the current trial, we will compare the DGDO strategy (control group) with a tapering strategy that combines DGDO with flare risk predictions (intervention group).

**Fig. 1:**
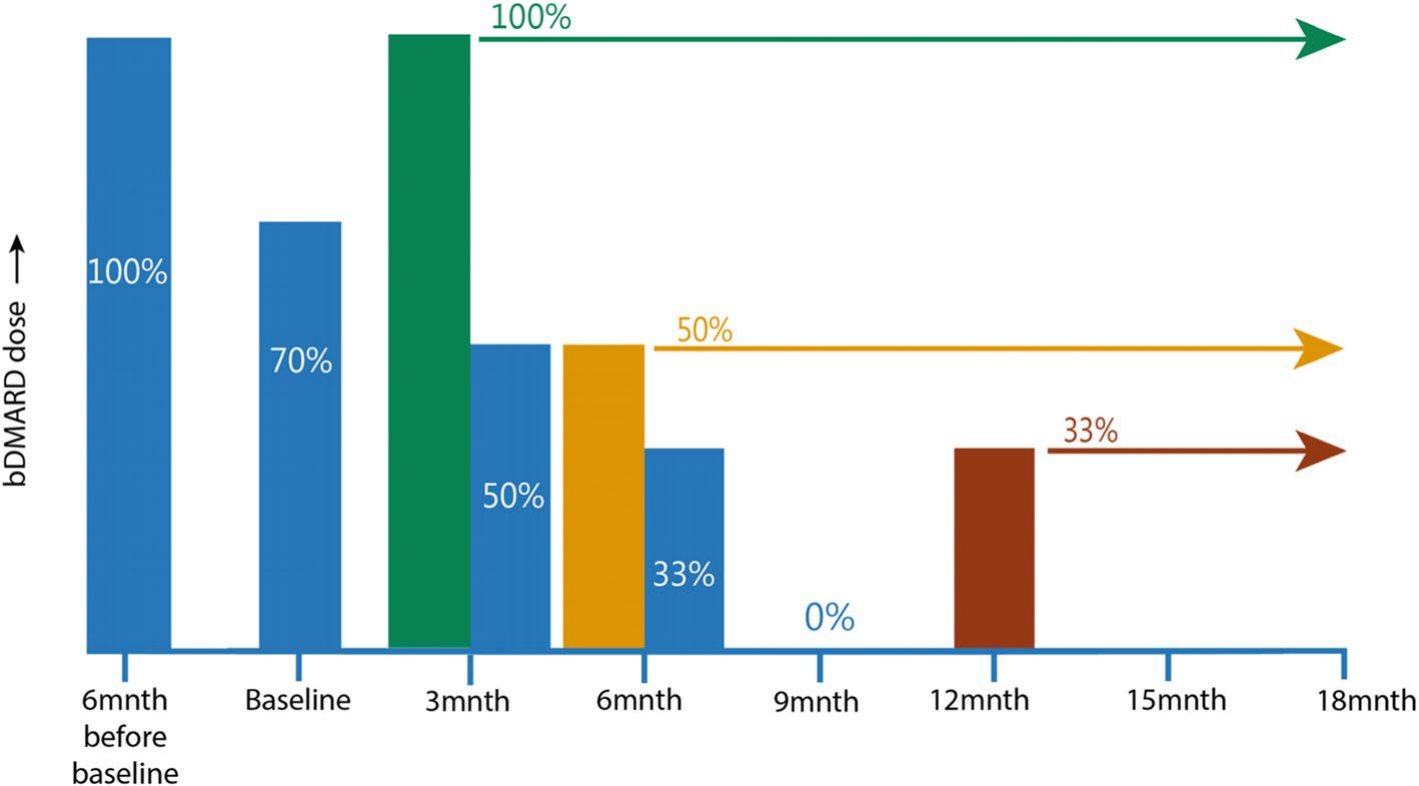
Tapering schedule. The *x*-axis shows the time in months. The *y*-axis shows the bDMARD dose expressed as percentage of the defined daily dose [12]. At every study visit, it will be assessed if tapering should be continued. The following are several examples, where the stated colors correspond to the colors of the bars in the figure. Standard tapering scheme (both groups, blue bars): if no flares occur and there is no high predicted risk of flare, then the bDMARD is tapered from 100% and discontinued at 9 months until the end of the study. Example 1 (both groups, green bar): the patient tapered until 70%. At the 3-month visit, a flare occurs. The patient increases the dose to 100% and remains at this dose until the end of the study. Example 2 (intervention group, yellow bar): the patient tapered until 50%. At the 6-month visit, there is a high predicted risk of a flare. The patient does not taper further and remains at 50% until the end of the study. Example 3 (intervention group, red bar): the patient tapered until 0%. At the 12-month visit, there is a high predicted risk of a flare. The patient increases the dose to 33% and remains at this dose until the end of this study

In the control arm of our trial (DGDO alone), the bDMARD is tapered stepwise every 3 months until the patient has a flare or until the bDMARD is discontinued. The tapering steps are defined as a rounded percentage of defined daily dose in the following order: 100%—70%—50% -33%—0% (Fig. 1). The defined daily dose is based on the Dutch national guidelines of standard dosages [12]. We chose this 4-step tapering approach rather than the 3-step tapering approach as used in DRESS [6] as this may reduce the risk of (severe) flaring in both groups [17], increases the number of decision steps where the prediction model can be applied, and is in line with other tapering studies [5]. Patients that have already made a tapering step and enter the study at ~ 70% (minimum 66%) of defined daily dose will follow the same schedule, with the exception of the first step (70%—50%—33%—0%).

Tapering is done for most bDMARDs by increasing the administration interval, rather than the administration dose. This is because (1) for some bDMARDs, lower dosages are not available (e.g., prefilled syringes of some bDMARDs only come in one dosage); (2) lower dosages can be flat priced and thus more expensive per mg (e.g., sarilumab 150 mg); and (3) this approach reduces the burden for the patient by reducing the amount of injections. The only exception to this rule is intravenously administered infliximab, as this already has a long standard dosing interval of 8 weeks. Further increasing this interval could reduce pharmacological efficacy [18] and would cause a dosing interval greater than the 3-monthly study visit interval. Dosing intervals are rounded to 0.5 weeks for feasibility reasons.

In case of a flare, the bDMARD dose is increased to the last effective dose, for both the control group and the intervention group. In addition, short-term glucocorticoids are allowed within the protocol. Flares are defined by an increase in DAS28-CRP of > 1.2 or an increase of > 0.6 where the resulting DAS28-CRP is > 2.9. At each study visit, the DAS28-CRP will be compared with baseline, rather than the last visit, to prevent undertreatment of patients with a gradual increase of disease activity. When a flare occurs, no further tapering attempts will be taken during the follow-up period. If a flare persists during 3 months, the bDMARD is increased to full dose. If a flare persists at the full dose of the bDMARD, further treatment is at the discretion of the treating rheumatologist.

If a patient experiences symptoms of a flare, the patient will be encouraged to plan an extra (i.e., unscheduled) study visit (USV). During an USV, no further tapering steps will be taken.

#### Intervention description {11a}

At each 3-monthly study visit, it will be assessed whether the tapering schedule as described above can be continued. In the intervention group, tapering is continued until a flare occurs (similar to the control group) *or* until there is a high predicted risk of a flare occurring in the next 3 months when taking the next tapering step.

The cut-off for a high predicted risk of flare is >  = 35%, determined as optimal in the simulation of clinical impact of the prediction model [11]. In case of a high predicted risk of a flare, the bDMARD is kept at the same dose, and no further tapering attempts are taken (Fig. 1). If the bDMARD is already discontinued and there is a high predicted risk of flare, the bDMARD will be restarted at 33% of the defined daily dose. The reasons for this exception are twofold. Firstly, the model predicts the risk of a flare when taking the next tapering step. For example, if there is a high predicted risk at the step from 50 to 33%, it will be advised to remain at 50% of the defined daily dose. But when the bDMARD is discontinued, the current dose and the next tapering step are however equal (both 0%), thus justifying a dose increase to 33%. Secondly, from a clinical perspective, it was deemed undesirable for both rheumatologists and patients to keep a previously stopped bDMARD discontinued when there is a high predicted risk of a flare, especially in the case of bDMARD monotherapy.

#### Criteria for discontinuing or modifying allocated interventions {11b}

If the treating physician (together with the patient) determines the treatment advice will not be followed this will be registered together with the reason for deviating from the advice.

#### Strategies to improve adherence to interventions {11c}

For the purpose of this study, a web-based dashboard has been developed that will be used in both the control group and the intervention group to facilitate adherence to the treatment protocol. This dashboard displays the disease activity (including the current presence of a flare) and DMARD use over time throughout the study, as well as the treatment advice specific for each patient. Rheumatologists are encouraged and trained to adhere to the treatment protocol, unless medical reasons require a deviation. These deviations must be registered in the study database. Furthermore, at each visit we will register whether the patient adhered to the treatment advice of the previous visit.

#### Relevant concomitant care permitted or prohibited during the trial {11d}

If a patient uses conventional synthetic (cs)DMARDs at screening, these should be continued at a stable dose during the study. An exception is made for short-term (max. 2 weeks) oral prednisone use to treat a flare to a maximum of 10 mg. The treating physician is allowed to deviate in any way from this treatment protocol if deemed medically necessary. Reasons for deviations from the protocol, csDMARD, glucocorticoid, and NSAID use will be registered throughout the duration of the study.

#### Provisions for post-trial care {30}

As both the control group and the intervention group will be treated with bDMARDs in dosages that are currently common in clinical practice, we do not expect (post-trial) harm from participation in the study. There are no specific provisions for post-trial care. Post-trial treatment is at the discretion of the treating rheumatologist.

### Outcomes {12}

#### Primary outcome

As we aim to determine whether incorporating flare risk predictions in a bDMARD tapering strategy can reduce flares during tapering to the lowest effective dose, our primary outcome is the number of flares per patient during the follow-up period of 18 months.

A flare is defined in line with previously validated definitions as follows [14, 15, 19]:

Compared to DAS28-CRP at baseline.

- An increase in DAS28-CRP > 1.2 or
- An increase in DAS28-CRP > 0.6, where the resulting DAS28-CRP > 2.9

Both compared to DAS28-CRP at baseline (i.e., before start of tapering).

### Secondary outcomes

To determine the clinical benefit of the incorporation of flare risk predictions in a bDMARD tapering strategy, the number of flares per patient must be interpreted together with the bDMARD dose reduction. If the addition of our flare prediction model is able to reduce flares, but does not reduce the bDMARD dose, it is of no use in a tapering strategy. Furthermore, the consequences of possible undertreatment or overtreatment with bDMARDs should be taken into account. These include the patient’s quality of life and adverse events related to either bDMARDs or disease activity.

- Clinical outcome measures
  - ◦ Presence of any (one or more) flare during the study
  - ◦ Presence of any major flare during the study (flare duration > 12 weeks)
  - ◦ DAS28-CRP over time
  - ◦ Mean bDMARD dose reduction, expressed as percentage of defined daily dose over 18 months (monthly full dose equivalents)
  - ◦ Use (dose and duration) of anti-rheumatic drugs other than bDMARDs during the study period
- Patient-reported outcomes

Measured every 3 months:

- ◦ Functional disability using the Health Assessment Questionnaire (HAQ DIv2 Dutch version) [20]
- ◦ Quality of Life using the EQ5D5L [21]
- ◦ Provider assessed general disease activity (GDA) on a visual analog scale (VAS)
- ◦ Patient assessment of pain on a VAS 0–100 mm
- ◦ Patient Acceptable Symptom State (PASS) [22]
- ◦ 7 scale Likert transition question [23], assessing the change in symptoms compared to the last study visit

Measured every month and in case of symptoms of a flare:

- ◦ Flare severity score: OMERACT RA Flare questionnaire [24]

Measured at the last study visit:

-
  - ◦ Patient satisfaction with treatment (SAPS) [25]
  - ◦ Physician satisfaction with care on a VAS 0–100 mm

- Safety outcomes
  - ◦ Infections for which antibiotic, anti-viral treatment or antimycotic therapy is prescribed
  - ◦ (Serious) Adverse events (probably or definitively) related to the bDMARD as assessed by the treating physician other than infections using the Rheumatology Common Toxicity Criteria [26]
  - ◦ (Serious) Adverse events related to increased disease activity due to tapering other than joint complaints and DAS28-CRP-based flares as assessed by the treating physician
- Other
  - ◦ Tapering attempted (yes/no) up to flare/high-risk of flare or discontinuation of the bDMARD
  - ◦ Proportion of clinical visits where treatment advice is not followed
  - ◦ Reasons for not following the advised treatment steps in both arms

#### Cost-effectiveness

Observed anti-rheumatic drug use and visits to the rheumatology outpatient clinic will be recorded. Direct medical (e.g., general practitioner visits) and nonmedical (e.g., travel expenses) costs as well as indirect costs (e.g., productivity loss) will be obtained using a Health Care Utilization and Work Productivity Questionnaire.

#### General characteristics

We will collect the following general patient characteristics at baseline:

- Demographic data: sex, age height and weight, level of education
- Smoking status (current, ever, never), pack years and alcohol use (current units/day)
- Medical history: year of RA diagnosis, rheumatoid factor and anti-CCP positivity, the Charlson Comorbidity Index [27]
- Anti-rheumatic treatment: current use of anti-rheumatic treatment, including dose and frequency of b/csDMARDs, glucocorticoids, and NSAIDs. Previous DMARD use will also be recorded.

### Participant timeline {13}

The participant timeline is shown is Table 1.

**Table 1:** Overview of participant timeline. *CRP* C-reactive protein, *EQ-5D-5L* questionnaire assessing quality of life, *GDA* global disease activity, *HAQ* health assessment questionnaire, *PASS* patient acceptable symptom state, *RA* rheumatoid arthritis, *SJC* swollen joint count, *TJC* tender joint count, *VAS* visual analog scale 0–100 mm

Visit number	V0 Screening	V1 Baseline	V2	V3	V4	V5	V6	V7	USV
Month	** − 3 to 0**	**0**	**3**	**6**	**9**	**12**	**15**	**18**	**…**
Informed consent	X								
Eligibility criteria	X								
Demographic data		X							
Medical history	X								
Anti-rheumatic treatment	X	X	X	X	X	X	X	X	X
Smoking and alcohol use		X							
Height, Weight		X							
Randomization		X							
Clinical disease parameters									
28TJC and 28SJC	X	X	X	X	X	X	X	X	X
Provider VAS GDA		X	X	X	X	X	X	X	X
Patient VAS GDA	X	X	X	X	X	X	X	X	X
Questionnaires									
HAQ-DI		X	X	X	X	X	X	X	
EQ-5D-5L		X	X	X	X	X	X	X	
RA flare questionnaire^a^		X	X	X	X	X	X	X	X*
PASS		X	X	X	X	X	X	X	X*
Likert transition question		X	X	X	X	X	X	X	X*
Health care utilization and work participation		X	X	X	X	X	X	X	
Patients satisfaction with care (Dutch SAPS)								X	
Physician satisfaction with care (VAS)								X	
Laboratory assessments									
CRP		X	X	X	X	X	X	X	X
Safety/adverse events									
(Serious) Adverse events		X	X	X	X	X	X	X	X

*USV* Unscheduled visit. Patients with complaints of a flare will be encouraged to plan an unscheduled visit

^a^Patients will also be asked to fill out this questionnaire in between visits at 4-weekly intervals and if patients experience any disease related complaints

### Sample size {14}

Based on our simulation results, over the follow-up of the trial (18 months), a mean of about 1.2 flares per patient can be expected in the DGDO control group. The number of flares is estimated to reduce from 1.2 to 0.75 flares over 18 months when incorporating the flare predictions [11]. We thus expect a reduction in flares of about 38% (relative risk of 0.63).

Assuming a more conservative relative risk of 0.65 and a base flare rate of 1.2, using a sample size calculation for a Poisson regression analysis and the program G*Power version 3.1.9.2, 152 patients (76 per group) are needed to detect this difference with a power of 80% and a two-sided alpha of 0.05. Therefore, we will include 160 patients (80 per arm) in our study, taking possible loss to follow-up into account.

### Recruitment {15}

Several outpatient rheumatology clinics in the Netherlands (aim 7) will participate in this trial. We have planned an enrolment period of 18 months. As the inclusion criteria apply to a large proportion of RA patients, the recruitment is deemed feasible.

### Assignment of interventions: allocation

#### Sequence generation {16a}

Patients will be randomly assigned to either control or intervention group in a 1:1 ratio, stratified by center using random block sizes.

#### Concealment mechanism {16b}

Randomization will be performed by the validated randomization algorithm in the Castor electronic data capture (EDC) system. The exact randomization algorithm is unknown to any of the investigators, thereby ensuring allocation concealment.

#### Implementation {16c}

Patients will be enrolled by a research physician or nurse of the rheumatology departments of participating centers. All patients who give consent for participation and who fulfill the inclusion criteria will be randomized. The allocation sequence will be generated by Castor EDC.

### Assignment of interventions: blinding

#### Who will be blinded {17a}

This study will not be blinded. In the control group, the tapering process is halted when a flare occurs. In the intervention group, the tapering process is halted when a flare occurs *or* when there is a high predicted risk of flare. Therefore, if it is advised to halt the tapering process in the absence of a flare, it will be evident that this is due to a high predicted risk of flare. Blinding is thus not feasible in this study. The outcome measures will be assessed by an (unblinded) research physician or nurse and by the patient. These include (partly) objective measures, such as the bDMARD dose and the DAS28-CRP. Knowledge of the assigned group might influence the subjective outcome measures in the benefit of the intervention group. For this, we will investigate the subjective vs. objective components of the disease activity scores within both groups.

#### Procedure for unblinding if needed {17b}

Not applicable as this study is not blinded.

### Data collection and management

#### Plans for assessment and collection of outcomes {18a}

The clinical and safety outcome measures will be determined by a physician or research nurse of the rheumatology department and will be registered in Castor EDC. Participating physicians and nurses will be trained prior to the start of the study.

Flares are defined based on the validated measure as described by van der Maas et al. [19]. We have chosen for the validated measure of DAS28-CRP rather than DAS28-BSE as CRP levels are more sensitive to short-term changes in disease activity, and ESR can be more influenced by a number of unrelated factors [28, 29]. Patient-reported outcomes and questionnaires are collected either electronically via e-mail (preferred) or on paper [19–25].

#### Plans to promote participant retention and complete follow-up {18b}

Participating centers will regularly receive updates of trial progress and promotion/practical material to enhance inclusion and follow-up of participants. If patients do not follow the tapering steps in line with the treatment protocol, patients will remain in follow-up according to the protocol and reasons for deviations from the treatment protocol will be recorded.

#### Data management {19}

We created a data management plan in line with the General Data Protection Regulation, which can be viewed upon request. Data will be managed within the Castor EDC system. This is a secure cloud-based platform that contains automatic range checks, and study IDs are used to pseudonymize all data.

#### Confidentiality {27}

The type of data that is collected is in line with the General Data Protection Regulation. In Castor, study IDs are used to pseudonymize all data. The key to the study ID is safely kept by the (local) coordinating investigator in each participating center. Data will be stored for 15 years.

#### Plans for collection, laboratory evaluation, and storage of biological specimens for genetic or molecular analysis in this trial/future use {33}

Not applicable. No biological specimens will be stored.

## Statistical methods

### Statistical methods for primary and secondary outcomes {20a}

#### Primary endpoint

The number of flares over 18 months per patient will be compared (superiority testing) between strategies using Poisson regression as appropriate for a “count” outcome variable. Center (as stratification factor used in randomization), bDMARD line (1st, 2nd or > 2nd bDMARD), and baseline disease activity will be used as (prognostic) covariates in this analysis. The appropriateness of the assumption regarding the Poisson distribution will be checked before performing the analysis. In case overdispersion is present, alternative analysis methods like the use of a negative binomial model will be considered according to the (at that time) state of the art. This will be defined in a formal statistical analysis plan to be finalized before database lock.

The primary analysis will be performed on the intention to treat (ITT) population consisting of all patients who were randomized to one of the strategies. All tests of significance will be performed two-sided with *α* = 0.05.

#### Secondary endpoints

Binary secondary outcomes will be compared between strategies using logistic regression analysis. For secondary continuous outcomes over time, a mixed effects model will be used to account for clustering of measurements within patients over time. The secondary analyses will be corrected for the same covariates as stated in the description of the primary endpoint analysis and according to the ITT principle, with a secondary analysis in the per protocol population.

#### Interim analyses {21b}

No interim analyses will be performed. As the treatments in both study arms are within the range of usual care, we do not anticipate differences between the arms that warrant early cessation of the study due to detrimental effects to the participant.

#### Methods for additional analyses (e.g., subgroup analyses) {20b}

We will perform an exploratory subgroup analysis to compare the effect of the use of the flare risk predictions between patients using a TNF-inhibitor (TNFi) and patients using a different biological. This will also be tested using a modeling approach with an interaction term between the type of bDMARD (TNFi or non-TNFi) and the intervention-arm.

A trial-based economic evaluation as well as a budget impact analysis will be performed. A Dutch Healthcare perspective as well as a societal perspective will be used in these analyses. The analysis will be performed according to Dutch guidelines for economic evaluations [30]. Extensive sensitivity analyses regarding, e.g., the price of biological DMARDs will be performed.

All analyses will be further specified in the statistical analysis plan, which can be viewed upon request.

#### Methods in analysis to handle protocol non-adherence and any statistical methods to handle missing data {20c}

In the case that more than 10% of patients have missing outcome values, data will be imputed before analysis, using multiple imputation by chained equations with baseline characteristics and disease activity characteristics of previous study visits as predictor.

#### Plans to give access to the full protocol, participant level-data and statistical code {31c}

The trial protocol is available from the corresponding author on reasonable request. Data will be handled according to the FAIR principles. After completion of the study, metadata will be available upon request. For access to participant level-data and statistical code, an application can be submitted to the corresponding author which will be reviewed by the trial steering committee.

### Oversight and monitoring

#### Composition of the coordinating center and trial steering committee {5d}

The trial steering committee consists of PW, AB, JT, AM, and MM, as stated on the title page. Their responsibilities include agreement on the final protocol, reviewing progress of study and data collection and if necessary agreeing changes to the protocol or budget to facilitate the smooth execution of the study. The trial steering committee will discuss the progress of the trial at least twice a year and more often if necessary. In addition, they will actively search for new published data that may be relevant for this trial.

(Local) research physicians/nurses, (local) study coordinators, and (local) principal investigators are, together with the trial steering committee, responsible for running the trial day-to-day and providing organizational support.

#### Composition of the data monitoring committee, its role and reporting structure {21a}

A data management plan has been created in collaboration with the data manager of the coordinating center and is available upon request. As both arms of this study are within the spectrum of regular care, no data safety monitoring board is installed.

#### Adverse event reporting and harms {22}

Serious adverse events of special interest for bDMARD treatment (AESI) will be collected via Castor EDC and will be reported to the CCMO (Central Committee on Research Involving Human Subjects). All (serious) adverse events ((S)AEs) reported will be classified according to the Rheumatology Common Toxicity Criteria v.2.1 [26].

#### Frequency and plans for auditing trial conduct {23}

The study will be monitored by a central monitor of the University Medical Centre Utrecht, according to the monitoring plan in line with the guidelines of the Dutch Federation of University Medical Centres (NFU) [31],

#### Plans for communicating important protocol amendments to relevant parties (e.g., trial participants, ethical committees) {25}

Any modifications to the protocol which may impact on the conduct of the study, potential benefit of the patient or may affect patient safety, including changes of study objectives, study design, patient population, sample sizes, study procedures, or significant administrative aspects will require a formal amendment to the protocol. Such amendments will be agreed upon by the trial steering committee, need to be approved by the Ethics Committee prior to implementation and notified to the health authorities in accordance with local regulations. When applicable, amendments will be made to the registration in the Netherlands Trial Register [32].

#### Dissemination plans {31a}

Overall trial results will be communicated to participants and will be published in a scientific journal. There are no publication restrictions. Thereafter, the research team will proactively disseminate the results through (inter)national congresses and in appropriate recommendations and guidelines, among others by A.A. den Broeder, who is a member of the EULAR(European League Against Rheumatism) RA recommendations group.

## Discussion

The PATIO trial will be the first study to assess the effect of incorporating the results of a dynamic flare prediction model into a bDMARD tapering strategy. With this study, we will address two major challenges in modern day health care: improving (cost-)effectiveness of treatment and implementation of predictive algorithms in clinical practice. We will discuss these topics separately.

Over the past decades, the steep increase in health care costs has led to a growing interest in health care cost-effectiveness research. Tapering bDMARDs has the potential to increase cost-effectiveness by reducing medication costs, side effects, and patient burden while maintaining the same patient outcome as treatment with a full bDMARD dose. The currently available bDMARD tapering strategies, however, still give an increased risk of short-lived flares [6, 7]. The conflicting evidence regarding the impact of these flares on functional outcome and impact on quality of life [7, 9] may make physicians and patients hesitant to start the tapering process [33]. The implementation of the flare prediction model into a bDMARD tapering strategy has the potential to reduce the risk of a flare and may thus also increase the willingness to start bDMARD tapering.

The second challenge is the implementation of predictive algorithms in medicine. From the abundance of prediction models developed for health care purposes, very few are actually implemented in clinical practice [34]. This may be due several reasons, such as uncertainties on how to specifically use resulting predictions in care, the willingness of patients and physicians to trust these models, and the scarcity of studies providing evidence for the effectiveness of using such prediction models in clinical practice. In addition, technical requirements may be challenging, such as complying with the medical device regulations and the need for a user-friendly interface. The PATIO trial addresses all these challenges and may thus facilitate the safe and effective implementation of predictive algorithms in clinical practice.

We have previously demonstrated the potential of the flare prediction model to reduce the number of flares during bDMARD tapering a simulation study [11]. In the current randomized controlled trial, we will assess whether these results are maintained when actually using the flare prediction model in a bDMARD tapering strategy in clinical practice. In a future study, we will also address the views of both RA patients and rheumatologists on the implementation of predictive algorithms in clinical practice.

## Trial status

Open for inclusion. Start of recruitment: 28 October 2021. Expected end date recruitment: 28 April 2023.

## Supplementary Information

**Additional file 1.**

## Data Availability

Data will be handled according to the FAIR principles. After completion of the study, metadata will be available upon request. For access to participant level-data and statistical code, an application can be submitted to the corresponding author which will be reviewed by the trial steering committee.

## Abbreviations

ACR: American College of Rheumatology
AE: Adverse event
AESI: Adverse Event of Special Interest
bDMARD: Biological disease-modifying antirheumatic drug
csDMARD: Conventional synthetic disease-modifying antirheumatic drug
CCP: Cyclic citrullinated peptides
CRP: C-reactive protein
DAS28-(CRP): Disease activity Index assessing 28 joints. DAS28-CRP includes laboratory measurement of C-reactive protein
DGDO: Disease activity-guided dose optimization
EDC: Electronic data capture
EULAR: European League Against Rheumatism
EQ-5D-5L: Questionnaire for measuring health-related quality of life
GDA: Global Disease Activity
HAQ: Health Assessment Questionnaire
LDA: Low disease activity
NFU: Dutch Federation of University Medical Centres
NSAID: Non-steroidal anti-inflammatory drug
PASS: Patient acceptable symptom state
RA: Rheumatoid arthritis
RCT: Randomized controlled trial
RF: Rheumatoid factor
SAE: Serious adverse event
SAPS: Patient satisfaction with care
SJC: Swollen joint count
TJC: Tender joint count
TNF-i: Tumor necrosis factor inhibitor
UMCU: University Medical Centre Utrecht
USV: Unexpected study visit
VAS: Visual analog scale
ZonMW: The Netherlands Organisation for Health Research and Development
