# Supplementary material for: Prediction Aided Tapering In rheumatoid arthritis patients treated with biOlogicals (PATIO): protocol for a randomized controlled trial
Source: Trials. 2022 Jun 16;23:494. doi: 10.1186/s13063-022-06471-x (PMC9202120; doi:10.1186/s13063-022-06471-x)
Supplement: Supplementary file 1 — Additional file 1. [file 13063_2022_6471_MOESM1_ESM.docx]

**Appendices**

Appendix A.

| **Drug** | **Standard dose definition** |
| --- | --- |
| Infliximab | IV: 3mg/kg / 8 weeks |
|  | s.c.: 120mg / 2 weeks |
| Etanercept | s.c.: 50mg / week |
| Golimumab | s.c.: 50mg / 4 weeks |
| Certolizumab | s.c.: 200mg / 2 weeks |
| Adalimumab | s.c.: 40mg / 2 weeks |
| Sarilumab | s.c.: 200mg / 2 weeks |
| Tocilizumab | IV: 8mg / kg per 4 weeks, with a maximum of 800mg |
|  | s.c.: 162 mg / week |
| Abatacept | IV: < 60 kg: 500 mg / 4 weeks  60–100 kg: 750 mg / 4 weeks  > 100 kg: 1000 mg / 4 weeks |
|  | s.c.: 125 mg / week |

IV = intravenous

s.c. = subcutaneous
